# Bilateral Bifid mandibular canals – Special relevance for a general dental practitioner. Report of 2 cases

**DOI:** 10.4317/jced.55575

**Published:** 2019-03-01

**Authors:** Amar Sholapurkar, Cailin Davies

**Affiliations:** 1 BDS, MDS, FAGE, (PhD). Department Head – Oral Radiology section. Lecturer in Clinical Dentistry and Oral Radiology. Radiation Safety officer and Possession Licensee. College of Medicine and Dentistry. James Cook University. PO Box 6811. Cairns. Queensland – 4870; 2 BDS. College of Medicine and Dentistry, James Cook University. Smithfield, Queensland - 4878. Australia

## Abstract

Dental practitioners may encounter bifid mandibular canals upon radiographic assessment, and may not understand the true extent of this presentation. The presentation of a bifid mandibular canal poses difficulties when carrying out dental procedures, including administration of mandibular block anaesthesia, oral surgical procedures of the lower third molars, orthognathic surgery and implant placement. Therefore it is of paramount importance that general dental practitioners be aware of the radiographic appearance, as incorrect diagnosis of this rare presentation can lead to detrimental consequences. The purpose of the communication is to draw dental practitioner’s attention to the variations of bifid mandibular canals and ensure practitioners interpret radiograph correctly to make an informed diagnosis and eliminate the risk of damage and discomfort in the event of surgical treatment being carried out. It is advised that dental practitioners refer for further imaging such as a cone beam scan for greater understanding of the anatomical variation.

** Key words:**Bifid mandibular canal, inferior alveolar nerve, bilateral bifid mandibular canal.

## Introduction

Several studies have presented with unilateral bifid canals, however to current understanding only few authors (1-4). have reported on the presence of bilateral bifid canals. Radiographically the mandibular canal appears as a dark linear shadow situated between two radiopaque thin borders superiorly and inferiorly as a result of lamellar bone surrounding the canal (5,6). Although anatomically the mandibular canal is known to be a single bilateral structure, variations such as bifid mandibular canals have been reported on back to 1973 (2,5-7). Several literature based reports by numerous authors have noted on the existence of bifid mandibular canals in the Panoramic radiographs (7). Nortje *et al.* (2,7-9) recognised the incidence of bifid mandibular canals as 0.9%, Grover and Lorton (7,9) found the incidence to be 0.08% whilst Langlais *et al.* (7,9) found 0.95% respectively. However the most recent report and findings in 2015 by Kalantar (5) found the incidence to be 1.2%.

Panoramic radiographs aid practitioners to identify variations in normal mandibular canal anatomy to ultimately prevent misinterpretation (5). Radiographically the unusual presentation shows the cortical radiopaque borders of the canals joining together to form a triangular area of bone; with separation of the canals represented by the vertex of the triangle (7). It should be however noted that the presence of a bifid canal cannot be identified in the buccal-lingual dimension due to superimposition (5,7). Sometimes, false double-canals can appear on the Panoramic radiograph due to the imprint of the mylohyoid nerve on the internal mandibular surface, where it travels to the floor of the mouth following separation from the inferior alveolar nerve (7,9). A cone beam computed tomography (CBCT) scan is an essential radiographic tool and it is strongly advised that dental practitioners refer patients for a scan to appropriately diagnose true bifid mandibular canals (10,11).

## Case Report

**Figure 1:**
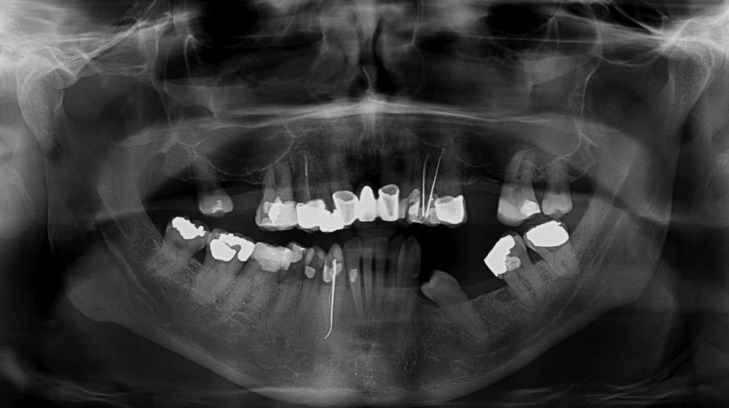
Case 1 - Bilateral bifid mandibular canal in Panoramic radiograph.

**Figure 2:**
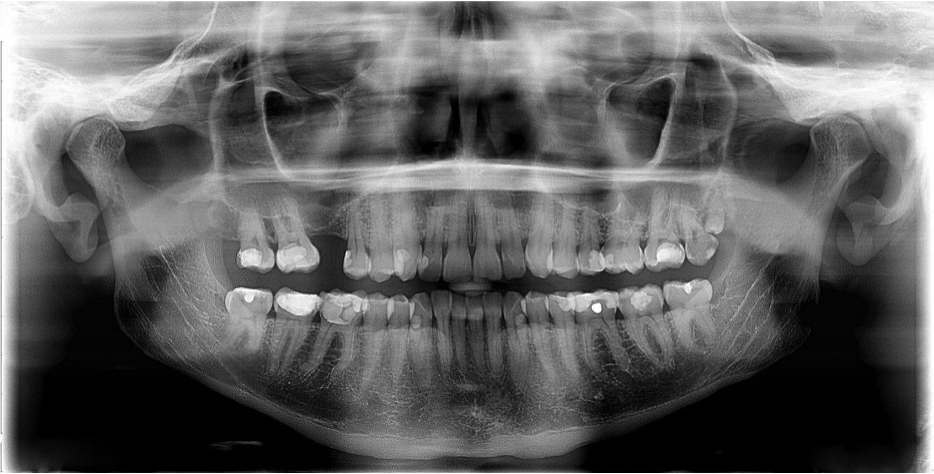
Case 2 - Bilateral bifid mandibular canal in Panoramic radiograph.

A 63-year old male patient, reported to the Dental clinic with multiple missing teeth who requested a replacement with fixed prosthesis. Panoramic radiograph was advised which revealed an incidental finding of a bilateral bifid mandibular canal (Fig. 1). To the best of our knowledge and current literature search revealed only 4 earlier references on this variation (1-4). In these cases the bilateral canals led to two separate mental foramen. It is evident in the case at hand that the bilateral bifid canals appeared to separate into two branches from the original mandibular canal and led to the same mental foramen on either side (3). In our case there was evidence of a bilateral bifurcation with varied morphology of the mandibular canal. The site of bifurcation on the left hand side could be visualised at the height of an imaginary line drawn horizontally along the occlusal plane at the height of the adjacent third molar. However the bifurcation site on the right hand side appeared to separate much lower in the mandibular ramus of the mandible. Our second case was a 37-year old male patient who reported to the Dental clinic and was curious to know if an implant could be placed in the region of upper right 1st molar region. Panoramic radiograph was advised which also revealed an incidental finding of a bilateral bifid mandibular canal (Fig. 2). The site of bifurcation on the left hand side was almost similar to our first case. However determination of the bifurcation site on the right hand side was more difficult and it appeared to separate much higher in the mandibular ramus of the mandible.

## Discussion

Panoramic radiographs detect mandibular canals in a two dimensional aspect, however when anatomical variations are present, depicting bi-dimensional images can be challenging (10). Dental practitioners should be familiar with other forms of radiographic technology to assist in understanding the extent of the anatomical variation (10,11). A CBCT scan is an additional radiographic assessment tool that practitioners can request, to aid in distinguishing between true and false bifid mandibular canals, CBCTs are required for correct diagnosis and superior visualisation of the canal to dental structures, surgical procedures should not be carried out without appropriate radiographic assessment (2,7,9-13). Our patients were lost for follow up and due to financial constraints CBCT was not considered.

Cross sectional CBCT provide dental practitioners important information regarding the mandibular canal and its precise trajectory, which allows for the precise localisation of the duplicated canal to eliminate the risk of potential nerve injury (6). A CBCT has the three dimensional ability to diagnose medio-laterally superimposed canals that cannot be visualised on panoramic radiographs, whilst scanning the entire trajectory of the main canal along with the associated branches being easily identifiable (2,5-7,9-11).

Carter and Keen (11) developed a classification on mandibular canals to determine the specific type of bifurcation based on their anatomical presentation (6,11). Type I is where the inferior alveolar nerve is represented a single large structure lying in a bony canal, type II where the inferior alveolar nerve is situated substantially lower down in the mandible and type III is where the inferior alveolar nerve is separated posteriorly into two large branches; type III has a direct relationship to the bifid mandibular canal variation (2,5,6,11). The classification of the canal in both the cases at hand were a type III relationship.

Bifid mandibular canals require much attention prior to surgical planning, understanding the location and configuration of mandibular canal variations is important for preoperative planning to alter and modify the procedure and take extra precautionary measures to prevent extensive damage to the nerve and surrounding tissues (2,6,13).

The most common problem that arises with the presence of a bifid mandibular canal is inadequate local anaesthesia (2,14). Administration of an IAN block is the most renowned method for mandibular anaesthesia, it is widely recognised that profound anaesthesia of half the mandible is not sometimes achieved (2,14). The cause of inadequate local anaesthesia is predominately due to error in operator technique or the presence of an anatomical issue. Conventionally anaesthesia of the ipsilateral chin, lip and teeth is indicative of an effective IAN block. If the patient experiences anaesthesia of the soft tissues around the injection site and still has sensation to the ipsilateral lips and chin, then the problem is related to operator technique, however if there is soft tissue anaesthesia of the lips, chin but not the teeth, practitioners should consider this to be an anatomical issue and further investigations should be carried out (2,12,14). Alternative methods of achieving appropriate anaesthesia can be through different injection techniques including, buccal and lingual infiltrations, Gow-Gates mandibular nerve block or the Vazirani-Akinosi closed mouth mandibular block technique (2,6,14).

Dental practitioners need to undertake third molar extractions and surgical procedures in the molar area with a bifurcation with caution as it is possible to have a second neurovascular bundle which has the potential to be damaged during extraction and invasive dental procedures, causing paraesthesia, dysesthesia, anaesthesia, neuroma development and can lead to significant bleeding following the procedure (6,11-13).

Implants are another surgical procedure carried out by dentists and other dental specialists, with inadequate insight of the three-dimensional aspect of the anatomy of the jaw/canal, the IAN can be traumatised by penetration of the drill preceding implant placement (13). This condition can also cause pain and discomfort in those patients who wear a dental prosthesis due to the bone resorption in the lower jaw (15).

Once anatomical variation has been detected, the practitioner can alter local anaesthetic technique and preoperative design for surgical procedures in order to reduce pain, discomfort and ultimately damage to the affected area (6,9,11-13).

## Conclusions

Bifid mandibular canals are too commonly unrecognised by practitioners on standard panoramic radiographs, this anatomical variation is of significant importance for surgical procedures and it is of utmost importance that dental practitioners are well educated in their presentation and be able to correctly diagnose this variation to enable adequate surgical planning in the region. It is strongly recommended when practitioners come across what is believed to be a true bifid canal that a CBCT is taken as these radiographs allow for greater accuracy in the diagnosis of anatomical variations in the jaw which reduces the incidence of nerve injury and damage to the neurovascular bundle.
